# Supplementary material for: Economic Burden of Cervical and Head and Neck Cancer in Taiwan from a Societal Perspective
Source: Int J Environ Res Public Health. 2023 Feb 20;20(4):3717. doi: 10.3390/ijerph20043717 (PMC9967678; doi:10.3390/ijerph20043717)
Supplement: Supplementary file 1 [file ijerph-20-03717-s001.zip › ijerph-2096446-supplementary.pdf]

Table S1. The ICD-9-CM, ICD-10-CM and ICD-O-3 definition for CC an HNC in the direct cost analysis

**Direct cost** Cancer type	ICD-9-CM	ICD-10-CM	ICD-O-3
Cervical Cancer	180.0–180.9	C53.0-C53.9	C53
Head and neck Cancer	141.0 and 141.6, 145.3-145.4, 146.0-146.9, 149.0-149.1	C01.9, C02.4, C02.8, C05.1, C05.2, C09.0, C09.1, C09.8, C09.9, C10.0, C10.1, C10.2, C10.3, C10.4, C10.8, C10.9, C14.0, C14.2 and C14.8	C00-06,C09-C10, C12-14

Table S2. The ICD-9 and ICD-10 definition for CC an HNC in the indirect cost analysis

**Indirect Cost**	2008~ (ICD 10)	~2007 (ICD9)
Cervical cancer	C53	180
Head and neck cancer	C00-C14, C30-C32	140-149, 160, 161

Table S3 The labor participation rate, unemployment rate and median annual income in Taiwan in 2019

2019 Age group	Labor participation rate	Unemployment rate	Annual income, median
<20	9.8%	9.22%	350000 NTD
20-24	58.84%	12.27%	
25-29	93.98%	6.57%	472000 NTD
30-34	92.81%	3.22%	534000 NTD
35-39	89.16%	3.18%	
40-44	83.55%	2.79%	568000 NTD
45-49	84.71%	1.99%	
50-54	74.43%	2.02%	534000 NTD
55-59	56.08%	1.65%	
60-64	36.70%	2.14%	

References:

1. Ministry fo Labor of Taiwan. *Labor Force Participation*; Ministry fo Labor of Taiwan: Taipei City, Taiwan, 2019.
2. Ministry of Labor of Taiwan. *Unemployment Rate*; Ministry fo Labor of Taiwan: Taipei City, Taiwan, 2019.
3. Directorate-General of Budget of Taiwan. *Earnings Exploration & Information System*; Directorate-General of Budget of Taiwan: Taipei City, Taiwan, 2019.
